# Unraveling the Antioxidant, Binding and Health-Protecting Properties of Phenolic Compounds of Beers with Main Human Serum Proteins: In Vitro and In Silico Approaches †

**DOI:** 10.3390/molecules25214962

**Published:** 2020-10-27

**Authors:** Raja Mohamed Beema Shafreen, Selvaraj Alagu Lakshmi, Shunmugiah Karutha Pandian, Yong Seo Park, Young Mo Kim, Paweł Paśko, Joseph Deutsch, Elena Katrich, Shela Gorinstein

**Affiliations:** 1 Department of Biotechnology, Alagappa University, Science Campus, Karaikudi, Tamil Nadu 630003, India; beema.shafreen@gmail.com (R.M.B.S.); lakshmivinay.317@gmail.com (S.A.L.); sk_pandian@rediffmail.com (S.K.P.); 2 Department of Horticultural Science, Mokpo National University, Muan, Jeonnam 534-729, Korea; ypark@mokpo.ac.kr; 3 Department of Food Nutrition, Gwangju Health University, Gwangsan-gu, Gwangju 506-723, Korea; bliss0816@hanmail.net; 4 Department of Food Chemistry and Nutrition, Jagiellonian University Medical College, Krakow 30-688, Poland; paskopaw@poczta.fm; 5 Institute for Drug Research, School of Pharmacy, Faculty of Medicine, The Hebrew University of Jerusalem, Jerusalem 9112001, Israel; josephd@ekmd.huji.ac.il (J.D.); ekatrich@gmail.com (E.K.)

**Keywords:** beer, phenolic compounds, antioxidants, binding, health properties, docking

## Abstract

Our recently published in vivo studies and growing evidence suggest that moderate consumption of beer possesses several health benefits, including antioxidant and cardiovascular effects. Although beer contains phenolic acids and flavonoids as the major composition, and upon consumption, the levels of major components increase in the blood, there is no report on how these beer components interact with main human serum proteins. Thus, to address the interaction potential between beer components and human serum proteins, the present study primarily aims to investigate the components of beer from different industrial sources as well as their mode of interaction through in silico analysis. The contents of the bioactive compounds, antioxidant capacities and their influence on binding properties of the main serum proteins in human metabolism (human serum albumin (HSA), plasma circulation fibrinogen (PCF), C-reactive protein (CRP) and glutathione peroxidase 3 (GPX3)) were studied. In vitro and in silico studies indicated that phenolic substances presented in beer interact with the key regions of the proteins to enhance their antioxidant and health properties. We hypothesize that moderate consumption of beer could be beneficial for patients suffering from coronary artery disease (CAD) and other health advantages by regulating the serum proteins.

## 1. Introduction

Beer is an important beverage, containing high amounts of polyphenols and showing antioxidant activity [1,2,3,4]. The phenolic compounds vary in high and low fermented, non-alcoholic and fruit beers [5,6,7]. It is known from a large number of reports that beer positively influences the health properties of human metabolism for protection from cardiovascular risk, lipid metabolism and antioxidant activity [8,9,10,11]. These actions depend on the antioxidant and anti-inflammatory properties of non-alcoholic compounds and slightly on the ethanol-dependent activity of beer [12,13]. Beer represents a source of phenolic compounds that could act synergistically, providing valuable data for moderate dietary beer inclusion studies [14,15,16,17]. The antioxidant properties of phenolics are responsible for the inhibition of oxidation of low density lipoprotein cholesterol. Moderate consumption of beverages in cholesterol-containing diets leads to a decrease in the content of total cholesterol in the liver in experiments on laboratory animals and in hypercholesterolemic patients [8,10,11,12]. Flavonoids could be linked to the beneficial effects of beer, as shown for the first time by our international research group in a number of reports in vitro and in vivo [9,18,19]. Recent studies have suggested that those flavonoids and some phenolic acids, which are abundant in beers, are present in many natural products and show health and binding properties [20,21]. Although numerous human studies have shown consistent effects of beer and other beverages on several intermediate markers for cardiovascular diseases [9,19,22,23,24], it is still unknown whether their action could be specifically related to polyphenols and especially to main human proteins (human serum albumin (HSA), plasma circulation fibrinogen (PCF), C-reactive protein (CRP), glutathione peroxidase 3 (GPX3)), which are relatively new biomarkers of coronary artery disease (CAD). In connection with the recent information described above, the present study aims to unveil the antioxidant capacities of phenolic compounds (total polyphenols, phenolic acids, flavonoids and flavanols), which are present in commercially available lager alcoholic beers in the context of health promotions, by in silico and in vitro analyses. The binding properties of investigated beers were determined in in vitro studies by fluorescence assays in comparison with main flavonoids and phenolic acids. Interactive behavior of the main serum proteins HSA, PCF, CRP and GPX3 with catechin, epicatechin, quercetin, ferulic and caffeic acids was also studied through molecular docking evaluation.

## 2. Results and Discussion

### 2.1. Total Polyphenols, Flavonoids, Flavanols and Phenolic Acids Content of Beers

The amounts of total polyphenols, flavonoids and flavanols in 11 beer samples are shown in Table 1.

On the basis of our published in vivo in results of health properties of moderate beer consumption [9,19,22,23], the main aim of the present study was to determine the functional properties of some individual phenolic compounds by interaction with the main human serum proteins, using fluorescence and molecular docking. Non-selective spectrometric methods were used for determination of several phenolic substances. A correlation was found between the most phenolic compounds, antioxidant and binding properties of beers. The comparison between the advanced analytical methods for determination of phenolic compounds was not the aim of this study, and in the literature there are numerous reports describing the analysis of these compounds and some of them were cited [6,7,16,25]. There are some differences and similarities in the obtained results. Total phenolic contents of low fermented lager beers were slightly lower in comparison with the previous report [5], showing the range of 373–473 mg/L of tyrosol (302–383 mg gallic acid equivalent (GAE)/L) of low fermentation of samples. The results of Amstel beer (Table 1) were higher than previously reported [5]. High fermentation beers showed a slightly higher amount of polyphenols from 453 to 599 mg/L of tyrosol (366.9–485.2 mg GAE/L), and only ‘Murphys’ showed 915 mg/L of tyrosol (741.2 mg GAE/L). In the Nardini et al. [6] study, the conventional lager beers showed lower polyphenol content (320.6–273.8 mg GAE/L) and total flavonoids (27–64 mg catechin equivalent (CE)/L) than in the investigated samples (Table 1, 668.3–442.1 mg GAE/L; 35.8–52.5 mg CE/L). The obtained results of total polyphenols were between 464.3 and 539.5 mg/L GAE. Chiva-Blanch et al. [10] evaluated that the amount of polyphenols in Carlsberg (510.2 ± 15.5 mg GAE/L) was higher in comparison with the values shown in Table 1 (450.5 ± 7.5 mg GAE/L). Oppositely, in the report of Mitić et al. [16], polyphenols in Amstel and Heineken beers were 1.46 and 1.11 times lower, respectively, than in Table 1. In the study of Mitić et al. [16], the amount of flavonoids in quercetin equivalent (QE) (103.9–185.3 mg QE/L) were relatively high and did not correlate with the values of the antioxidant activities of Amstel and Heineken beers. These results differed from those presented in the previous report [26], where the total polyphenols of Maccabee beer were 345.1 ± 12.1 mg GAE/L, epicatechin −65.5 mg/L and quercetin −0.95 mg/L. The amount of total flavanols in the presently measured samples did not show direct correlation between total polyphenols and antioxidant activities (Table 1). Beer contains a complex mixture of phenolic compounds (hydroxybenzoic acids (gallic acid), hydroxycinnamic acids (ferulic acid) and flavonoids (catechin)) that have expressed high antioxidant activity [3]. It was shown as well that caffeic acid is found in the lowest concentrations than other phenolic acids, and ferulic acid and some flavonoids were the most abundant [1,3,16,17], and therefore, in investigated beers, individual phenolic compounds were determined (Table 2). The correlation between the highest (Goldstar (GOLD), Kamenitza (KAM), Rostocker (ROST)), average (Maccabee (MACC), Heineken (HEIN), Oranjeboom (ORJB), Amstel (AMST), Żywiec (ŻYW)) and the lowest (Carlsberg (CARL), Miller Genuine Draft (MGD), Corona (COR)) concentrations of total polyphenols and flavonoids (Table 1) and the amounts of caffeic and ferulic acids, catechin, epicatechin and quercetin (Table 2) was found.

The obtained results differ from other reports, where slightly lower estimations of ferulic acid (0.85–2.16 mg/L), catechin (0.57–1.21 mg/L) and epicatechin (0.08–0.39 mg/L) were reported [16,17,25] than determined (Table 2). Most of the reports showed that among the different phenolic acids, ferulic and gallic acids are the most copious in commercial beers (around 14 and 6 mg/mL, respectively), followed by sinapic, vanillic, caffeic, *p*-coumaric, syringic and 4-hydroxyphenylacetic acids (between 0.5 and 4.2 mg/mL) [2,6]. Gallic and ferulic acids were more than 50% of the total content of individual phenolic compounds found during beer studies and are the most reported phenolics in beer [1,3]. The comparison of the same type of beer, but produced in different countries, showed differences because of the modifications in the technological processes, raw materials and conditions of the extraction of the main components. According to the data presented in the report of Szwajgier [17], the total amounts of phenolic acids in Heineken and Corona beers were 6.78 ± 0.39 and 6.13 ± 0.43, respectively. These results differ from the ones presented in Table 2. As was shown in the same report [17], vanillic and ferulic acids exerted a lower share of total antiradical activity against free radicals than the minor phenolic acids; therefore, caffeic acid was determined in all investigated beer samples (Table 2).

### 2.2. Beer Antioxidant Activities

The antioxidant activities of investigated beers are presented in Table 1. The obtained results were higher than reported by Nardini and Foddai [6], where the antioxidant activities of lager beers varied and showed values by 2-azino-bis (3-ethyl-benzothiazoline-6-sulfonic acid) diammonium salt (ABTS) assay in the range of 1.5–1.8 mM Trolox equivalent (TE) in comparison with the data in Table 1 (1.8–2.7 mM TE). The present results were in accordance with the published report of Habschied et al. [25], where three different kinds of lager beers (4.7–5.2% (*v*/*v*) of alcohol content) showed corresponding values of ABTS tests of 1.29–2.03 mM TE/L. Low antioxidant values of beer samples such as 0.21–0.23 mM TE were reported by Mitić et al. [16]. It can be concluded that the antioxidant activities measured in conventional beers varied, but were consistent with our previous results and with the published data [2,9,26,27,28,29]. The results of the β-carotene test were in correlation with the values of the ABTS assay (Table 1). The obtained results of some investigated beers can be compared with the report of Wang et al. [28]. In this report, Heineken beer showed the amount of total polyphenols of 393.9 mg GAE/L and the corresponding ability to scavenge free radicals by 1,1-diphenyl-2-picrylhydrazyl (DPPH) assay, of 27%. The same type of beer (Table 1) produced in another country showed the amount of total polyphenols of 466.3 mg GAE/L and the ability to scavenge free radicals, using β-carotene assay, with scavenging activity of 25%. Corona beer with total polyphenols of 285 mg GAE/L and the ability to scavenge free radicals (DPPH scavenging activity of 21%) can be compared with the same type of beer in which the amount of total polyphenols was 442 mg GAE/L. The ability to scavenge free radicals by the β-carotene test was similar to the published results [28] and showed scavenging activity of 24% (Table 1). The values of the polyphenols, flavonoids and the two antioxidant assays, and the expression of the units of antioxidant activities, did not change the correlation of the presented indices in all investigated industrial beer samples. The correlation with ABTS assay was slightly higher than with the β-carotene test, suggesting that ABTS test is based on hydrogen-donating ability. This fact underlines that phenolic compounds mainly influence the antioxidant properties of beer. It is also suggested that the beer samples with relatively high ABTS values can stabilize active oxygen radicals and have better flavor stability [3,6].

### 2.3. Binding Properties of Beers and Some Phenolic Compounds with Main Human Proteins

The binding properties of beer samples and some individual phenolic compounds were compared in interaction with human serum albumin and plasma circulation fibrinogen (Table 3).

HSA had a strong fluorescence emission peak at 343 nm, when excited with a wavelength of 280 nm. The addition of beer samples and pure phenolic compounds caused a gradual decrease in the fluorescence intensity of HSA, and the emission maximum had a red-shift of 8 nm. The principles of such measures and the obtained results (Table 3) are documented in the report of Poloni et al. [20], who used the classic indirect method of fluorescent quenching of tryptophan residues for the binding of polyphenols with porcine LDL and BSA, and where the binding data were obtained by titration of the proteins with increasing amounts of phenolic ligands. In this way, Stern Volmer plots have been obtained, and this allowed the measurement of binding constants and determination of the static nature of quenching, and the inner filter effects were negligible at the phenols concentrations used. In the present report, we have used a simplified measure to show only the decrease of fluorescence emission after the addition of a single concentration of ligands. This can be regarded as a relative measure of binding, providing that the inner filter is similarly negligible within the series of ligands. Thus, the % decrease of fluorescence represents the fraction of the binding sites of the protein occupied by the ligand, rather than the fraction of the total ligand bound to the protein (Table 3). The same report [20] showed the results of the experimental binding study using fluorescent quenching for quercetin and its 3-O-glucuronide. The albumin binding site for polyphenols had been previously identified by Dufour and Dangles [21]. Pattanayak et al. [30] and Latruffe et al. [31] reported binding properties between ellagic acid, resveratrol and other polyphenols, where phenolic acids and flavonoids effectively quenched the intrinsic fluorescence of HSA by static quenching. Leontowicz et al. [32] and Kim et al. [33] showed the binding properties of polyphenols from kiwi fruit and persimmons with HSA. All the above studies, including the present one, showed the evaluation of transport and releasing efficiency at the target site in the human physiological system since HSA is the most important carrier protein in blood serum. Our explanation of the obtained data was based on the interaction of the polyphenols and flavonoids with the main serum protein HSA. Oppositely, Poloni et al. [20] and Tung et al. [24] found that competition studies between serum albumin and LDL showed that substantial lipoprotein binding occurs even in the presence of a great molar excess of albumin, the major blood protein. The excitation of fibrinogen gave an emission maximum at 344 nm, which had a shift of 6 nm with the binding of phenolic acids and some beer samples. As can be seen from the results (Table 3), the obtained evaluation is in agreement with recent reports about the influence of ethanol with HSA and fibrinogen interaction, where the binding was in the range of 2.6–3.1%. According to some reports [32,33], the ethanolic extracts showed quenching of HSA in comparison with water extracts of about 2.9%. Ethanol has a low influence on the quenching of HSA, but in different samples of investigated beers, having high amounts of total polyphenols and flavonoids (Table 1), increasing binding percentages appeared (Table 3, binding of ROST about 31%), and corresponded to higher antioxidant activity of the product. These results are in agreement with the data reported in [34], where the efficiency of flavonoids as free radical scavengers was proved. The obtained results (Table 3) on quenching of fibrinogen with investigated samples (12.5–18.1%) are in line with other reports, where the absorption peak at about 351 nm was measured at the interaction of resveratrol, and the fluorescence intensity exhibited a decrease [35,36,37]. The comparison of the obtained results of quenching of HSA with the investigated samples showed about 1.7 times higher quenching than with fibrinogen, especially with flavonoids, and this is in agreement with a recent report [37]. As it was shown in Table 1, Table 2 and Table 3, there is a correlation between polyphenols, antioxidant activities and binding properties of the investigated beer samples. The low values of ethanol binding with HSA and fibrinogen once more supports the hypothesis that it is not alcohol that prevents coronary artery disease, but rather the non-alcoholic composition of beer which contains a high amount of phenolic substances [38]. In the study of Sierksma et al. [38], plasma C-reactive protein and fibrinogen levels were decreased after three weeks’ consumption of beer, as compared to non-alcohol beer consumption. The conclusion of this report is that moderate alcohol consumption significantly decreased these two indices, based on anti-inflammatory action of alcohol in the protection of coronary artery disease. These conclusions are opposite to the present report and to our previous results in in vitro and in vivo studies [19,22,26]. In the present study results showed that binding properties of main human serum proteins with ethanol are not the main components in beer. Oppositely, the non-alcoholic substances prevent CAD, which was proved also by molecular docking evaluation. It is impossible to compare the pure compounds found in beer with real beer samples. The obtained binding properties of the pure standards and the beer samples are not equal (Table 3). The results are dependent on synergism of the bioactive substances in the product. From another point of view [24], it was found that low plasma concentrations make polyphenols and their metabolites poor plasma antioxidants. The concentration of these compounds in lipoproteins and cells is sufficient for polyphenols to act in the protection of heart diseases using their antioxidant properties.

### 2.4. Molecular Docking of Beer Components with Serum Proteins

Molecular docking studies with CRP revealed that flavonoids have achieved high dock score >58 compared to the phenolic acids. The flavonoids, epicatechin and quercetin, with the dock score of 60.268 and 58.609, respectively, have shown a similar binding pattern to CRP (Table 4).

**Figure 1:**
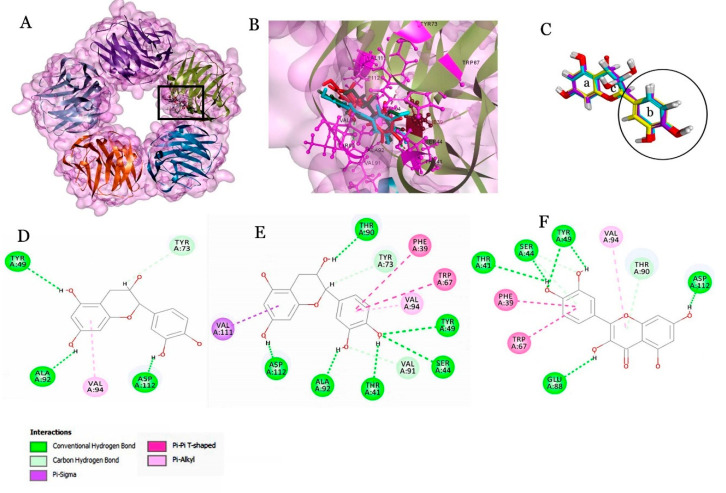
Molecular docking studies with C-reactive protein (CRP). (**A**) Interaction of the ligands into binding pocket (black box) of the pentameric protein; (**B**) expanded view of the binding pocket shows the interacting amino acids (ball and stick model) with the ligands; (**C**) molecular overlay of the flavonoids—catechin, epicatechin and quercetin (a, b) aromatic ring and c is the heterocyclic ring. Circle represents the aromatic ring (b) which has favored the interaction with CRP. (**D**–**F**) represent the 2D view for interaction of catechin, epicatechin and quercetin with CRP.

The most common amino acids showing interaction with epicatechin and quercetin are PHE39, THR41, SER44, TYR49, TRP67, THR90, VAL94 and ASP112 (Figure 1).

**Figure 2:**
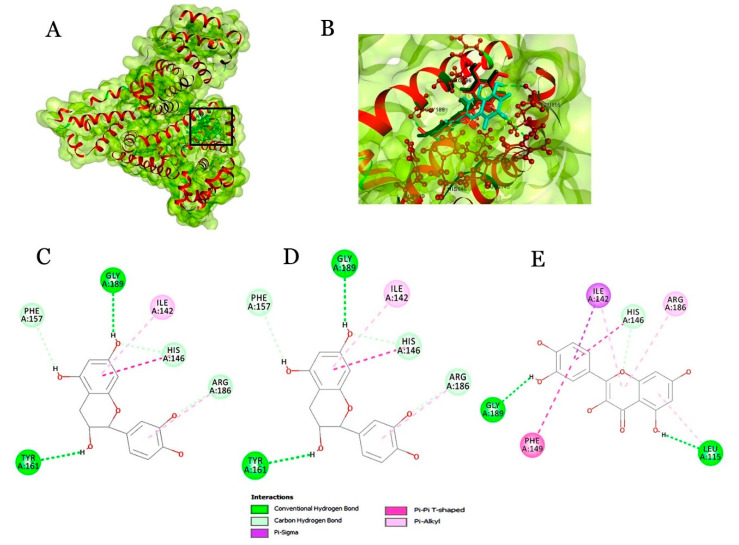
In silico docking of HSA. (**A**) Interaction of the ligands into binding pocket (black box) of HSA; (**B**) Expanded view representing ligands interacting with the amino acids of the receptors (ball and stick model); 2D plot for interaction of catechin (**C**), epicatechin (**D**) and quercetin (**E**) with HSA.

From HSA docking analysis, both flavonoids and phenolic acids had interactions in domain I, the major drug binding pocket of HSA. The crucial residues involved in binding are ILE142, HIS146, PHE149, TYR161, ARG186, GLY189 and LEU115. Among these, TYR161 are the crucial residues involved in drug recognition (Figure 2). All the three flavonoids investigated in the study have achieved the highest dock score >50. Catechin, epicatechin and quercetin have also shown consistent interaction with the key residues (TYR161). However, the phenolic acids have shown a dock score of >35. In the case of GPX3, epicatechin exhibited the highest dock score of 103.36, followed by quercetin with a dock score of 102.45 (a score identical to the dock score of epicatechin). Phenolic acids have similar dock scores, 82.448 and 83.955 for ferulic and caffeic acids, respectively.

**Figure 3:**
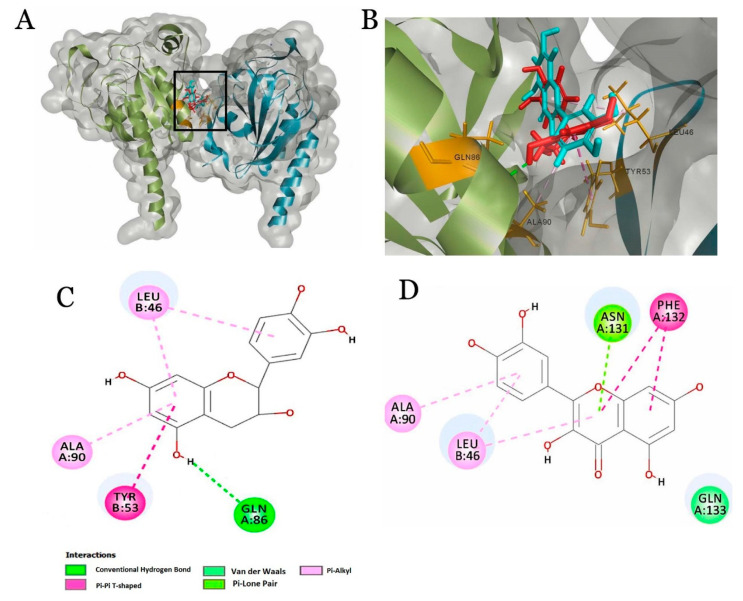
Interaction analysis with glutathione peroxidase 3 (GPX3) protein. (**A**) The black box represents the binding pocket of the tetrameric protein. (**B**) Expanded view shows ligand interaction with the amino acids in the binding pocket; 2D plot representing the amino acid interaction with epicatechin (**C**) and quercetin (**D**).

Overall, flavonoids show the highest dock score compared to the phenolic compounds and the residues implicated in the interactions were LEU46, TYR53, GLN86, ALA90, ASN131, PHE132 and GLN133 (Figure 3).

**Figure 4:**
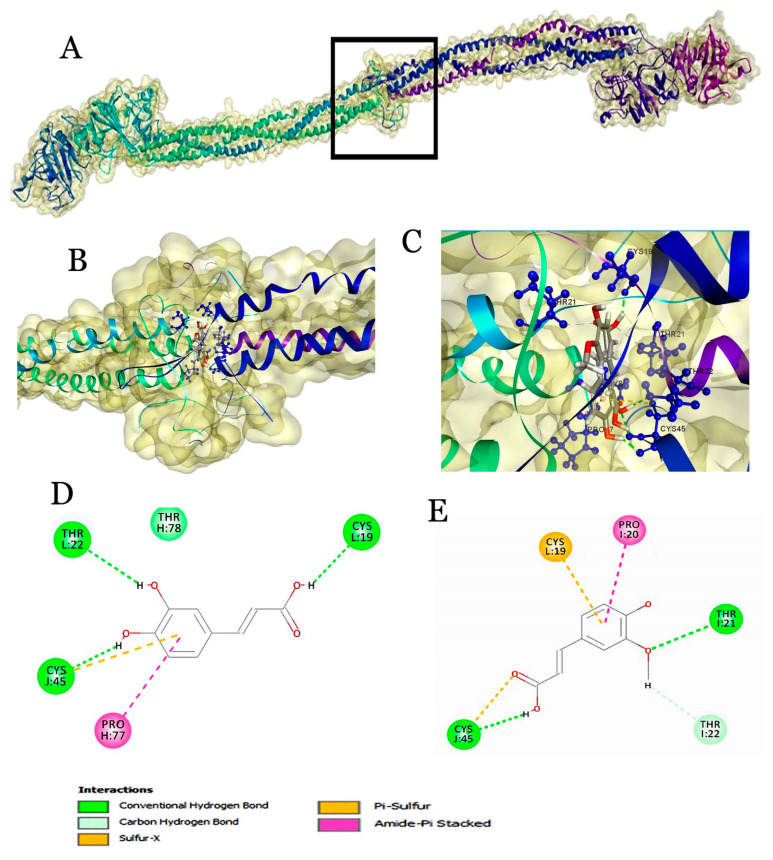
Molecular docking of ligands with fibrinogen. (**A**) Surface view of fibrinogen representing the central nodule (black box) present in the E region; (**B**) Expanded view of the central nodule. (**C**) Interaction of ligands with the binding pocket amino acids of fibrinogen; 2D plot showing interaction with caffeic acid (**D**) and ferulic acid (**E**).

Fibrinogen interaction with beer components revealed the possible interactions with phenolic acids with the dock score of 95.517 and 95.094 for ferulic acid and caffeic acid, respectively. The residues such as CYS19, PRO20, THR21, THR22, CYS45, THR78 and PRO77 are involved in interactions (Figure 4).

As it was mentioned previously, the secondary metabolites such as flavonoids and phenolic acids, which are found as well in beer, are investigated widely as antioxidants to prevent oxidative damage responsible for many diseases such as cancer, atherosclerosis, dyslipidaemia, chronic inflammation and other diseases [39,40,41,42]. Flavonoids and phenolic acids are well-known for their therapeutic benefits but as candidates their effectiveness still remains unclear. In the present study, the flavonoids and phenolic acids from the beer were investigated for their interactive behavior with serum proteins such as C-reactive protein (CRP), human serum albumin (HSA), GPX3 and fibrinogen through molecular docking studies.

Among the target proteins, CRP is a known biomarker detected in the human serum during inflammation as well as classified as a putative pattern recognition receptor (PPR) of the innate immune system, which indicates the invasion of the pathogens and removal of dead cells by eliciting the innate complement pathway [43,44,45]. CRP activates the macrophages and induces oxidative stress damage; therefore, CRP is also regarded to be itself a risk factor for cardiovascular diseases. The amount of CRP determines the risk levels of different diseases and is an indicator for cardiovascular disease (CVD), rheumatoid arthritis (RA), lupus nephritis and chronic inflammation. Moua et al. [46] reported that coffee, containing bioactive compounds, may reduce CRP levels as a biomarker of chronic inflammation. Mangnus et al. [47] showed that moderate alcohol consumption is protective against RA development and associated with lower levels of systemic inflammation in RA and with lower levels of CRP. However, autoantibodies are produced against 35–47 amino acids of CRP which is associated with the severity of the disease [48]. Thus, the epitope interacts with CRP after undergoing a conformational change.

Additionally, the residues covering from 35–47 amino acids are considered to be the important residues for therapeutics and diagnostics studies. Among them, LEU37, PHE39, TYR40 and LEU43 amino acids were found buried in the native protein and exposed only when the protein underwent conformational change of the monomeric form as epitope. Epicatechin and quercetin interacting with key residues of CRP is determined to be significant for therapeutic studies. Though catechin and epicatechin are under the same class flavan-3-ol, the binding pattern with CRP is completely different. The difference in catechin and epicatechin is mainly due to the presence of the hydroxyl groups in the β and α position of the C3, respectively (Figure 1C). Quercetin is a flavanol, and its chemical structure completely lacks the OH group in the C3 position. However, quercetin has shown similar interaction as epicatechin (flavan-3-ol) with CRP. In addition, epicatechin and quercetin were reported [30,34,44] as important dietary flavonoids with strong antioxidant properties and were investigated for their preventive role against CVD. HSA is an important biomarker which is synthesized in liver and found circulating in the blood. HSA has an indispensable role as an important antioxidant of blood and maintains the blood pH level. Besides, HSA is regarded as an important carrier for exogenous and endogenous substances. HSA also plays an important role in pharmaceuticals by binding to the drug and preventing the oxidation of the drug. However, a low level of HSA indicates the risk level of cardiovascular disease [49]. On the contrary, HSA is associated with an anti-inflammatory role, but the mechanism is unclear.

HSA is a 67 kDa protein with 585 amino acid residues. It consists of three identical domains (5–190, 191–383 and 384–585) with two drug binding sites, I and II. Site I appears at the second domain while site II appears at the third domain. Interestingly, docking of HSA with beer components revealed the interactions at the rearmost end of the first domain.

GPX3, a selenium containing glutathione peroxidase 3, is synthesized in the kidney and actively expressed in plasma. It protects the cells from oxidative stress by catalyzing the hydrogen peroxide into alcohol [50]. It has already been reported that flavonoids, in particular quercetin, interacts with GPX through in vitro studies. Besides the antioxidant potential of the quercetin–GPX complex, it has also been reported for cytotoxicity effect. However, there are no clear reports on how flavonoids bind to GPX3 at the molecular level. Here, docking with beer components revealed that flavonoids such as quercetin and epicatechin have a higher affinity toward GPX3 (than polyphenolic acids) and this observation was well-consistent with the previous in vitro report of Nagata et al. [51], wherein they have shown the interaction of endogenous GPX with flavonoids in rat BL9 (hepatocyte) cells through in vitro assays.

Additionally, they have shown that synergistic interaction of flavonoids and GPX are critical factors for enhancing their antioxidant activities. Here, flavonoids exhibited good interactions with GPX3. Nonetheless, the residues that make interactions with GPX3 partially differ among them which indicates that each flavonoid may have a differential binding region. In the case of phenolic acids, the residues implicated in binding are similar, suggesting that phenolic acids may interact with GPX3 in a similar fashion. Surprisingly, the overall interactions were observed at the adjacent region to the active site residue Seu-73 of GPX3. But how these flavonoids binding alter the conformations of GPX3 to activate the enzymes requires a comprehensive study. Overall, our results suggest that these flavonoids interact in the distal region of the GPX3 active site and account for antioxidant potential in the plasma. Moreover, high levels of such interactions with GPX protein can enhance the GPX3 activity. This enhancement has a beneficial role in reducing the risk of cardiovascular and chronic kidney diseases [52]. It also delays the aging process as aging occurs mainly due to the decline in GPX3 [53]. Fibrinogen is a glycoprotein, which circulates in blood plasma and is synthesized by the liver [54,55,56]. It comprises two sets of non-identical polypeptide chains termed α, β and γ (α_2_β_2_γ_2_). An enzymatic conversion of fibrinogen into fibrin by thrombin is one of the critical steps for maintaining the homeostasis of blood. Here, ferulic and caffeic acids made interactions with all three α, β and γ chains (Table 4). When compared to flavonoids, phenolic acids (ferulic and caffeic acids) exerted higher affinity towards the central nodule of the E region of fibrinogen. In general, hydroxycinnamic acids are illustrious for antioxidant property. Thus, based on our results, we postulate that phenolic acids have an important role in interacting with fibrinogen than flavonoids.

The present results are in good agreement with the fluorescence measurements and literature report of Luo et al. [55], in which three type II phenolic acids (caffeic, *p*-hydroxycinnamic and ferulic acids) were used to synthesize a total of 18 phenolic acid derivatives. With molecular docking for molecule design and the evaluation of haemostatic and anticoagulant activities with blood assays, the data of Luo et al. [55] indicated that caffeic acid derivatives showed certain anticoagulant or procoagulant activities and that two other series contained compounds with the best anticoagulant activities (Table 4). The interaction of fibrinogen with investigated flavonoids and their docking is in line with other reports [56], where six compounds, including quercetin, catechin and epicatechin, were examined for the inhibition of thrombin amidolytic activity. Quercetin, catechin and epicatechin caused the inhibition of thrombin amidolytic activity and only quercetin from the three mentioned above changed thrombin proteolytic activity. It is possible that these compounds can change the activity of thrombin. From another point of view, most phenolic substances are not stable in vivo and their bioavailability in the digestive tract is relatively low. From a number of previous and present experiments (Table 3), it was proved that polyphenol compounds can also bind with many components of blood plasma (mainly by human serum albumin) and the real effect of these compounds on coagulation may be mediated also by a different mechanism than their action on thrombin [56].

As was mentioned previously, the present study was aimed at investigating the interaction of individual components with different serum proteins that are responsible for the health benefits. Overall, the study indicates that beer components such as flavonoids and phenolic acids interact with the key regions of the proteins to enhance their antioxidant and binding properties. Among them, flavonoids have a significant role in enhancing the beneficial properties. It has already been reported that consumption of beer increases the flavonoid and phenolic acid content in the plasma and thereby promotes the cardiovascular health benefits [8,9,10,11,12,18,19,22,24].

## 3. Materials and Methods

### 3.1. Materials

Caffeic and ferulic acids, catechin, epicatechin, quercetin, Trolox, human serum albumin, fibrinogen, sodium nitrite, aluminum chloride, potassium peroxodisulfate and 2,2′-azino-bis(3-ethylbenzothiazoline-6-sulfonic acid) diammonium salt (ABTS), were from Sigma (St. Louis, MO, USA). Standard phenolics were dissolved in methanol (1 mg/mL), stored at −80 °C.

### 3.2. Samples

Commercial beer bottles were purchased at markets and beer shops and were investigated in this study. The eleven beers were common lager beers from different countries of production (Maccabee (MACC); Goldstar (GOLD); Heineken (HEIN); Carlsberg (CARL); Miller Genuine Draft (MGD); Corona (COR); Oranjeboom (ORJB); Amstel (AMST); Kamenitza (KAM); Rostocker (ROST); Żywiec (ŻYW)). Every sample was bought in triplicate, from the same batch and with the identical shelf life. The sample set included craft and mainstream beer varieties with alcohol by volume ranging from 4.1 to 5.6%. Four beer samples were produced in Israel. Beer bottles were stored in the dark and analyzed immediately after opening. All beer samples were first degasified and then pH was adjusted to 7.0 before analysis with additions of an appropriate amount of 0.1 M sodium hydrogen phosphate solution. Separate samples from the same bottle were frozen at −80 °C for antioxidant status and bioactivity.

### 3.3. Analyses of Bioactive Compounds

The total polyphenols were determined by the Folin–Ciocalteu method [57], where beer samples were diluted with distilled water till 1 mL, then 0.1 mL of Folin–Ciocalteu’s reagent was added. After 5 min, 0.2 mL sodium carbonate (35% *w*/*v*) was added. Final volume was adjusted to 2 mL with distilled water. After 1 h in the dark, absorbance at 765 nm was measured against an appropriate blank reagent. The results were expressed as milligrams of gallic acid equivalents per liter of beer.

Total flavonoids were determined in 0.05 mL aliquots of the sample using the spectrophotometric method [58], where beer samples were diluted with distilled water to a final volume of 1.5 mL, and then 0.075 mL of 5% NaNO_2_ solution was added. After 6 min, 0.15 mL of 10% AlCl_3_ hexahydrate was added and allowed to stand for an additional 5 min, before 0.5 mL 1 M NaOH was added. The volume was adjusted to 2.5 mL with distilled water, mixed, and absorbance at 510 nm was measured immediately. The results are expressed as milligrams of catechin equivalents per liter of beer.

Total flavanols (TFLs) were estimated using the *p*-dimethylaminocinnamaldehyde (DMACA) method, where 0.2 mL of beer was introduced into a 1.5 mL Eppendorf tube, and 1 mL of DMACA solution was added. The mixture was vortexed and allowed to react at room temperature for 10 min. The absorbance at 640 nm was then read against a blank prepared similarly without DMACA. The presence of flavanols on the nuclei with subsequent staining with the DMACA reagent resulted in an intense blue coloration in beer [59].

Some phenolic acids (ferulic and caffeic) and flavonoids (catechin, epicatechin and quercetin) were determined with a HPLC system [3,6,13,60]. The phenolic compounds from beer samples were extracted according to the procedures, described by Nardini and Foddai [6], Bartolomé et al. [13] and Pozo-Bayon et al. [60]. A volume of 50 mL of each of 11 beer samples was extracted three times with 25 mL of diethyl ether and then three times with 25 mL of diethyl acetate, and the organic fractions were combined. After 30 min of drying with anhydrous Na_2_SO_4_, the extract was filtered through a Whatman-40 filter and evaporated to dryness in a rotary evaporator. The residue was dissolved in 2 mL of methanol/water (1:1, *v*/*v*) and analyzed by high-performance liquid chromatography (HPLC), according to the conditions described in the Bartolomé et al. [13] report. A Waters (Milford, MA, USA) chromatograph equipped with a 600-MS controller, a 717 plus autosampler and a 996 photodiode-array detector was used. A gradient of solvent A (water/acetic acid, 98:2, *v*/*v*) and solvent B (water/acetonitrile/acetic acid, 78:20:2, *v*/*v*/*v*) was applied to a reverse-phase Nova-pack C18 column (30 cm × 3.9 mm Internal Diameter (I. D.)), as following as follows: 0–55 min, 80% B linear, 1.1 mL/min; 55–57 min, 90% B linear, 1.2 mL/min; 57–70 min, 90% B isocratic, 1.2 mL/min; 70–80 min, 95% B linear, 1.2 mL/min; 80–90 min, 100% B linear, 1.2 mL/min; 90–120 min. For HPLC analysis, an aliquot (50 μL) was injected onto the column and eluted at the temperature of 20 °C. Samples were prepared and analyzed in duplicate.

### 3.4. Determination of Antioxidant Activities

The total antioxidant activity of beers was evaluated by the ABTS radical cation decolorization (ABTS) assay [61] and β-carotene bleaching assay on 0.01 mL of beer samples. The ABTS radical cation was formed by ABTS solution (7 mM) with potassium persulfate (2.45 mM) in distilled water at room temperature, for 16 h before use. A working solution (ABTS reagent) was diluted to obtain absorbance values of 0.7 at 734 nm and equilibrated at 30 °C. After addition of ABTS solution, the absorbance reading was taken 1 min after initial mixing, and up to 6 min percentage inhibition of absorbance was calculated with reference to a Trolox calibration curve and evaluated as mM Trolox equivalent/L of beer.

In an antioxidant assay using a β-carotene linoleate model system, 4 mL of emulsion containing β-carotene (0.2 mg) in 0.2 mL of chloroform, linoleic acid (20 mg) and Tween-40 (200 mg) was mixed, and then the chloroform was removed at 40 °C under vacuum. The resulting mixture was diluted with 10 mL of water. To this emulsion was added 40 mL of oxygenated water. The emulsion (4 mL) was added to the investigated sample. The absorbance at 470 nm was taken for 120 min at an interval of 20 min. The synthetic antioxidant butylated hydroxyanisole (BHA) in EtOH was used for comparative purposes and added to the sample. The antioxidant activity (AA) of the samples was evaluated in terms of bleaching of the β-carotene [62].

### 3.5. Fluorimetric Measurements

Two-dimensional fluorescence (2D-FL) measurements for all beer samples were recorded on a model FP-6500, Jasco spectrofluorometer, serial N261332, Tokyo, Japan, equipped with 1.0 cm quartz cells and a thermostat bath. The 2D-FL measurements were taken at emission wavelengths from 310 to 500 nm and at excitation of 295 nm. For comparison of the obtained results, caffeic and ferulic acids, catechin, epicatechin and quercetin were used [31]. The solutions for the reaction were in the following concentrations: 1.0 × 10^−5^ mol/L HSA; 0.05 mol/L Tris HCl buffer with 0.1 mol/L NaCl, pH 7.4. Fibrinogen stock solution was made by dissolving in phosphate buffer (10 mM, pH 7.4) to obtain a concentration of 20 μM. The initial fluorescence intensities of HSA and PCF were measured before the interaction with the investigated samples and pure substances and after interaction with the samples (quenching of fluorescence emission of proteins in our case of HSA and fibrinogen) upon addition of pure phenolic compounds or samples from beer. The differences of the measured fluorescence intensities were used for calculation of the relative binding properties, because the ligands were used only in one concentration, and the decrease of fluorescence represents the fraction of the binding sites of the protein by the ligand [20,32,33].

### 3.6. Molecular Docking Studies Using Main Human Serum Proteins

Crystal structures of human C-reactive protein (CRP) (PDB ID: 1B09), human serum albumin (HSA) (PDB ID: 1H9Z), human glutathione peroxidase 3 (GPX3) (PDB ID: 2R37) and human fibrinogen (PDB ID: 3GHG) with a resolution of 2.5 Å, 2.5 Å, 1.85 Å and 2.9 Å, respectively, was obtained in PDB format from the PDB database. Similarly, flavonoids and phenolic acids reported in the study were downloaded from the PubChem database in SDF format. The protein and ligand structures were minimized by applying a CHARMM force field and the spherical cut-off radius of 13.0 Å was set for non-bonded interaction. All other parameters were set to their defaults. The potential binding site region of the target proteins were determined using ‘Define and edit binding site’ protocol. The active site for CRP, HSA, GPX3 and fibrinogen was determined at site 1 (grid box, X: 142.694, Y: 153.060, Z: 30.358), site 1 (grid box, X: 37.172, Y: 10.895, Z: 13.554), site 1 (grid box, X: 21.467, Y: −1.923, Z: −13.674000) and site 5 (grid box, X: 103.163, Y: −40.380, Z: −92.422), respectively. The LigandFit module from Discovery Studio 2.5 (DS2.5) was used for performing the docking studies. Based on the scoring functions, the top scoring ligands resulting with best pose were extracted and analyzed through BIOVIA-DS 17 R2 client [63].

### 3.7. Statistical Analysis

All results were calculated as the mean with standard deviations. Comparison of the mean values was performed using Duncan’s Multiple Range Test. All analyses were performed in five replicates.

## 4. Conclusions

As the health promoting advantages of beer was mentioned previously in various reports, the present study was aimed at investigating the interaction of individual components of beer with different serum proteins that are responsible for health benefits. Overall, the study unveiled that beer components enriched with flavonoids and phenolic acids interact with the key regions of the serum proteins to enhance their antioxidant and binding properties. Among them, flavonoids had a significant role in enhancing the beneficial properties. It has already been reported that consumption of beer increases the flavonoid and phenolic acid content in the plasma and thereby promotes the cardiovascular health benefits. Our study unveiled that phenolic acids and flavonoids might exert an appreciable health benefit by making contact with serum proteins and significantly contribute to maintain the endogenous redox homeostasis in host. However, in the case of excessive beer consumption, how it exerts deleterious effects needs an elaborate study.

## Figures and Tables

**Table 1:** Antioxidant properties of beer samples.

Beer Code	Style	Country of Production	Alcohol Strength % Vol	Total Pol., mg GAE/L	Total Flavonoids, mg CE/L	Total Flavanols, mg CE/L	β-Carot, % AA	ABTS, mM TE
**MACC**	Pale lager	Israel	5.0	510.1 ± 10.1 ^b^	45.1 ± 0.5 ^b^	40.3 ± 1.8 ^a^	28.1 ± 0.8 ^b^	2.06 ± 0.01 ^b^
**GOLD**	Dark lager	Israel	4.9	552.6 ± 9.6 ^a,b^	48.9 ± 0.8 ^a,b^	23.3 ± 1.4 ^c^	30.7 ± 1.2 ^a,b^	2.21 ± 0.02 ^a,b^
**HEIN**	Pale lager	Israel	5.0	466.3 ± 6.2 ^c^	41.3 ± 0.7 ^c^	21.9 ± 1.5 ^d^	25.2 ± 0.7 ^c^	1.88 ± 0.02 ^c^
**CARL**	Pale lager	Israel	5.0	450.5 ± 5.5 ^c,d^	40.1 ± 0.6 ^c,d^	21.2 ± 0.9 ^d^	24.6 ± 0.8 ^d^	1.82 ± 0.01 ^c,d^
**MGD**	Pale lager	USA	4.6	456.7 ± 7.2 ^c,d^	40.8 ± 0.9 ^c,d^	16.3 ± 0.4 ^e^	25.1 ± 1.1 ^c^	1.85 ± 0.01 ^c,d^
**COR**	Pale lager	Mexico	4.5	442.1 ± 4.3 ^d^	35.8 ± 0.5 ^d^	19.2 ± 0.8 ^d,e^	24.2 ± 0.7 ^d^	1.79 ± 0.03 ^d^
**ORJB**	Pale lager	Netherlands	5.0	482.3 ± 6.8 ^b,c^	42.9 ± 0.9 ^b,c^	29.6 ± 1.5 ^b^	26.7 ± 1.0 ^b,c^	1.95 ± 0.01 ^b,c^
**AMST**	Pale lager	Netherlands	4.1	501.3 ± 7.5 ^b,c^	44.3 ± 1.3 ^b,c^	21.6 ± 0.9 ^d^	27.6 ± 1.1 ^b,c^	2.02 ± 0.01 ^b^
**KAM**	Pale lager	Bulgaria	4.4	647.4 ± 11.3 ^a^	51.1 ± 1.4 ^a^	25.4 ± 1.1 ^b,c^	33.6 ± 1.3 ^a^	2.61 ± 0.05 ^a^
**ROST**	Golden lager	Germany	4.9	668.3 ± 13.3 ^a^	52.5 ± 1.5 ^a^	26.4 ± 1.2 ^b,c^	34.5 ± 1.2 ^a^	2.68 ± 0.03 ^a^
**ŻYW**	Blond lager	Poland	5.6	471.3 ± 7.2 ^c^	41.5 ± 0.9 ^c^	22.2 ± 1.0 ^c,d^	26.6 ± 1.0 ^b,c^	1.90 ± 0.02 ^b,c^

Values are means ± SD of 5 measurements; Means within a column with the different superscripts are statistically different (*p* < 0.05; Student’s *t*-test). Abbreviations: Maccabee (MACC); Goldstar (GOLD); Heineken (HEIN); Carlsberg (CARL); Miller Genuine Draft (MGD); Corona (COR); Oranjeboom (ORJB); Amstel (AMST); Kamenitza (KAM); Rostocker (ROST); Żywiec (ŻYW); gallic acid equivalent (GAE); catechin equivalent (CE); total polyphenols (Total Pol.); 2,2′-azino-bis (3-ethylbenzothiazoline-6-sulfonic acid) assay (ABTS); Trolox equivalent (TE).

**Table 2:** Individual phenolic compounds of beer samples (mg/L).

Beer Code	Caffeic Acid	Ferulic Acid	Catechin	Epicatechin	Quercetin
**MACC**	2.17 ± 0.08 ^b,c^	14.10 ± 0.39 ^b^	3.03 ± 0.06 ^b^	1.09 ± 0.08 ^a,b^	1.40 ± 0.08 ^b^
**GOLD**	2.34 ± 0.07 ^b^	15.22 ± 0.54 ^a,b^	3.27 ± 0.09 ^a,b^	1.17 ± 0.07 ^a,b^	1.52 ± 012 ^a,b^
**HEIN**	1.97 ± 0.07 ^c,d^	12.92 ± 0.45 ^c,d^	2.78 ± 0.07 ^b,c^	0.99 ± 0.05 ^b,c^	1.24 ± 0.07 ^c^
**CARL**	1.91 ± 0.04 ^c,d^	12.48 ± 0.32 ^d^	2.69 ± 0.09 ^c^	0.96 ± 0.09 ^b,c^	1.28 ± 0.08 ^b,c^
**MGD**	1.94 ± 0.05 ^c,d^	12.62 ± 0.35 ^c,d^	2.69 ± 0.08 ^c^	0.97 ± 0.06 ^b,c^	1.25 ± 0.11 ^b,c^
**COR**	1.87 ± 0.06 ^d^	12.23 ± 0.44 ^d^	2.64 ± 0.08 ^c^	0.94 ± 0.07 ^c^	1.21 ± 0.07 ^c^
**ORJB**	2.07 ± 0.08 ^c^	13.31 ± 0.54 ^c^	2.83 ± 0.05 ^b,c^	1.02 ± 0.07 ^b^	1.32 ± 0.13 ^b^
**AMST**	2.12 ± 0.06 ^b,c^	13.89 ± 0.48 ^b,c^	2.99 ± 0.09 ^b,c^	1.07 ± 0.07 ^b^	1.37 ± 0.01 ^b^
**KAM**	2.73 ± 0.06 ^a^	17.87 ± 0.61 ^a^	3.82 ± 0.12 ^a^	1.37 ± 0.08 ^a^	1.77 ± 0.12 ^a^
**ROST**	2.83 ± 0.08 ^a^	18.47 ± 0.51 ^a^	3.98 ± 0.15 ^a^	1.42 ± 0.09 ^a^	1.83 ± 0.07 ^a^
**ŻYW**	2.08 ± 0.07 ^c^	13.01 ± 0.36 ^c^	2.26 ± 0.08 ^d^	1.01 ± 0.07 ^b^	1.29 ± 0.09 ^b,c^

Values are means ± SD of 5 measurements; Means within a column with the different superscripts are statistically different (*p* < 0.05; Student’s *t*-test). Abbreviations: Maccabee (MACC); Goldstar (GOLD); Heineken (HEIN); Carlsberg (CARL); Miller Genuine Draft (MGD); Corona (COR), Oranjeboom (ORJB); Amstel (AMST); Kamenitza (KAM); Rostocker (ROST); Żywiec (ŻYW).

**Table 3:** Binding properties of beer samples, standard flavonoids and phenolic acids with human serum proteins.

Beer Code	λ_em_ (nm)	FI (A.U.)	Binding to HSA (%)	λ_em_ (nm)	FI (A.U.)	Binding to PCF (%)
**MACC**	349	731.8 ± 2.1 ^c,d^	24.1 ± 2.5 ^b^	347	674.9 ± 2.8 ^d^	14.1 ± 0.9 ^c^
**GOLD**	350	713.6 ± 2.6 ^d^	26.0 ± 2.8 ^a,b^	348	666.3 ± 1.2 ^e^	15.1 ± 1.2 ^b^
**HEIN**	347	750.9 ± 3.8 ^b,c^	22.2 ± 2.7 ^c^	346	684.3 ± 2.7 ^c^	12.8 ± 0.8 ^d^
**CARL**	346	759.4 ± 3.5 ^b,c^	21.3 ± 2.6 ^c,d^	346	683.6 ± 2.8 ^c^	12.9 ± 1.1 ^d^
** MGD **	346	755.3 ± 4.2 ^b,c^	21.7 ± 1.9 ^c,d^	346	685.9 ± 2.1 ^c^	12.6 ± 0.9 ^d^
**COR**	345	763.9 ± 4.3 ^b,c^	20.8 ± 1.5 ^d^	346	686.7 ± 2.7 ^c^	12.5 ± 1.1 ^d^
** ORJB **	348	745.1 ± 5.8 ^c^	22.8 ± 1.9^c^	346	680.0 ± 3.0 ^c^	13.3 ± 1.2 ^c,d^
**AMST**	349	735.9 ± 5.5 ^c,d^	23.7 ± 1.3 ^b,c^	346	676.5 ± 4.1 ^d^	13.8 ± 1.1 ^c,d^
**KAM**	350	671.3 ± 6.3 ^e^	30.4 ± 1.4^a^	350	646.7 ± 4.3 ^f^	17.6 ± 1.5 ^a^
**ROST**	351	663.2 ± 7.3 ^e^	31.3 ± 1.5 ^a^	350	642.4 ± 4.2 ^f^	18.1 ± 1.3 ^a^
**ŻYW**	347	748.8 ± 4.2 ^c^	22.4 ± 0.9 ^c^	346	682.8 ± 4.0 ^c^	13.0 ± 0.9 ^c,d^
**EtOH**	344	934.8 ± 2.9 ^a^	3.1 ± 0.2 ^f^	343	764.3 ± 5.9 ^a,b^	2.6 ± 0.1 ^f^
**Catechin**	348	743.8 ± 4.4 ^c^	22.9 ± 1.9 ^c^	344	736.9 ± 5.7 ^b^	6.1 ± 0.7 ^e^
**Epicatechin**	348	745.7 ± 4.8 ^c^	22.7 ± 2.0 ^c^	344	738.7 ± 5.4 ^b^	6.9 ± 0.9 ^e^
**Quercetin**	347	754.4 ± 4.7 ^b,c^	21.8 ± 2.1 ^c,d^	344	743.2 ± 6.1 ^b^	5.3 ± 0.5 ^e,f^
**Caffeic acid**	345	820.1 ± 3.2 ^a,b^	14.9 ± 1.5 ^e^	360	667.9 ± 4.9 ^e^	14.9 ± 1.1 ^c^
**Ferulic acid**	345	803.6 ± 5.2 ^b^	16.7 ± 1.5 ^d,e^	360	663.9 ± 4.9 ^e^	15.4 ± 0.9 ^b^
**HSA/buffer**	343	964.7 ± 3.8 ^a^	-	-	-	-
**PCF/buffer**	-	-	-	344	784.8 ± 4.9 ^a^	-

Values are means ± SD of 5 measurements; Means within a column with the different superscripts are statistically different (*p* < 0.05; Student’s *t*-test). Abbreviations: Maccabee (MACC); Goldstar (GOLD); Heineken (HEIN); Carlsberg (CARL); Miller Genuine Draft (MGD); Corona (COR); Oranjeboom (ORJB); Amstel (AMST); Kamenitza (KAM); Rostocker (ROST); Żywiec (ŻYW); human serum albumin (HSA); maximum emission peak (λ_em_); fluorescence intensity (FI); arbitral units (A.U.); plasma circulation fibrinogen (PCF).; Binding to HSA (%) and binding to PCF (%) is the % decrease of fluorescence emission of the fractions of the binding sites of the proteins occupied by the ligand.

**Table 4:** Molecular docking results are indicated with dock score for the flavonoids and phenolic acids against different serum proteins.

Ligand Name	Dock Score	Bond Formation	Chain	Interacting Amino Acids
**Human C-Reactive Protein**				
**Catechin**	62.693	2(H-bond),1(Pi-sigma)	Chain A	ALA92, VAL94, ASP112
**Epicatechin**	60.268	3(H),1(Pi-alkyl), 1(carbon-H)	Chain A	PHE39, THR41, SER44, TYR49, TRP67, TYR73, THR90, VAL91, ALA92, VAL94, ASP112, VAL111
**Ferulic acid**	55.343	6(H),1(Pi-sigma),1(Pi-alkyl), 2(carbon-H)	Chain A	TYR49, TYR73, ALA92, VAL94, ASP112
**Caffeic acid**	53.062	2(H),1(Pi-alkyl),2(carbon-H)	Chain A	TYR73, VAL89, ALA92, VAL94, ASP112
**Quercetin**	58.609	6(H),2(Pi-Pi),1(Pi-alkyl),1(carbon-H)	Chain A	PHE39, THR41, SER44, TYR49, TRP67, THR90, GLU88, VAL94, ASP112
**Human Serum Albumin**				
**Catechin**	53.679	2(H),1(Pi-Pi),2(Pi-alkyl),3(carbon-H)	Chain A	ILE142,HIS146,PHE157,TYR161,ARG186, GLY189
**Epicatechin**	53.033	2(H),2(Pi-Pi),1(Pi-alkyl),1(Pi-sigma)	Chain A	ILE142, HIS146, PHE149, TYR161, ARG186, GLY189, LEU115
**Ferulic acid**	39.165	1(Pi-Pi),1(Pi-alkyl)	Chain A	ILE142, PHE157
**Caffeic acid**	36.825	3(Pi-Pi),1(Pi-alkyl),1(Van der Waals)	Chain A	ILE142, PHE157, HIS146, GLY189, LYS190
**Quercetin**	51.170	2(H),1(Pi-Pi),1(Pi-alkyl),1(carbon-H), 1(Pi-sigma)	Chain A	ILE142, HIS146, PHE149, TYR161, ARG186, GLY189
**Human glutathione peroxidase 3 (GPX3)**				
**Epicatechin**	103.364	2(H),1(Pi-Pi),3(Pi-alkyl)	Chain A	LEU46, TYR53, GLN86, ALA90
**Ferulic acid**	82.449	1(Pi-Pi),1(Pi-alkyl), 1(carbon-H)	Chain A	TYR53, ALA90, ASN131
**Caffeic acid**	83.956	1(Pi-Pi),1(Pi-alkyl), 1(carbon-H)	Chain A	TYR53, ALA90, ASN131
**Quercetin**	102.459	1(amide-Pi),2(Pi-alkyl), 1(Pi-lone), 1(Van der Waals)	Chain A	ALA90, ASN131, LEU46, PHE132, GLN133
**Human Fibrinogen**				
**Ferulic acid**	95.517	2(H),1(Pi-amide),1(Pi-S), 1(carbon-H)	Chain J (α), Chain I (β), chain L (γ)	CYS19, PRO20, THR21, THR22, CYS45
**Caffeic acid**	95.095	3(H),1(Pi-amide),1(Pi-S), 1(carbon-H),1(Van der Waals)	Chain J (α), Chain H (β), Chain L (γ)	CYS19, THR22, CYS45, THR78, PRO77
